# Immunonutrients for radiation-induced oral mucositis in head and neck cancer: current evidence and future perspectives

**DOI:** 10.3389/fimmu.2026.1885556

**Published:** 2026-07-28

**Authors:** Xingqing Long, Chunye Fang, Xuezhen Qiu, Fayi Yu, Libing Chen, Xunmei Liu, Yuxin Li, Yinghua Chen, Rensheng Wang, Tingting Zhang

**Affiliations:** 1 Department of Radiation Oncology, the First Affiliated Hospital of Guangxi Medical University, Nanning, China; 2 Guangxi Key Laboratory of Immunology and Metabolism for Liver Diseases, Nanning, China; 3 Key Laboratory of Early Prevention and Treatment for Regional High-Frequency Tumor (Guangxi Medical University), Ministry of Education, Nanning, China

**Keywords:** head and neck cancer, immunonutrients, nutritional support, radiation-induced oral mucositis, radiotherapy

## Abstract

Radiation-induced oral mucositis (RIOM) is one of the most common and severe complications in patients with head and neck malignancies undergoing radiotherapy or chemoradiotherapy, with an incidence of 80–100%. More than half of patients develop severe RIOM (grade 3–4), resulting in intense pain, dysphagia, and malnutrition, which significantly impairs quality of life. However, conventional nutritional support primarily provides basic nutrients and lacks immunomodulatory effects. Immunonutrition, characterized by a favorable safety profile and good tolerability, not only meets nutritional requirements but also modulates immune responses, attenuates inflammation, and supports mucosal barrier repair. It has been shown to enhance patients’ tolerance and response to radiotherapy and chemotherapy, thereby improving clinical outcomes. Given that inflammation, oxidative stress, and mucosal barrier disruption are central to the pathogenesis of RIOM, these properties suggest that immunonutrition may play a beneficial role in its prevention and management. This narrative review summarizes the current evidence on immunonutrition in RIOM and explores its potential as a therapeutic approach for its prevention and treatment, with the aim of providing useful insights for improving patient quality of life and clinical outcomes.

## 1 Introduction

Radiation-induced oral mucositis (RIOM) refers to inflammation, erosion, or ulcerative lesions of the oral mucosa that occur in patients with head and neck cancer (HNC) following radiotherapy (1), and it is one of the most common adverse effects of radiotherapy. Among HNC patients undergoing curative radiotherapy or chemoradiotherapy, the incidence of RIOM reaches as high as 80%–100% (2). During the acute phase of RIOM, patients experience pain, dysphagia, taste disorders, and dysarthria. Without effective management, these symptoms may lead to severe malnutrition over time. Prolonged nutritional deficiency further delays mucosal healing, increases the risk of infection, and creates a vicious cycle between tissue injury and impaired recovery (3). In severe cases, RIOM may even result in interruption of radiotherapy or chemoradiotherapy, ultimately compromising clinical outcomes (4). Previous studies have shown that early nutritional intervention can effectively improve the nutritional status and protein levels of HNC patients undergoing radiotherapy, thereby reducing the incidence of RIOM and promoting mucosal healing (5). However, conventional nutritional support mainly provides basic nutrients (such as proteins, carbohydrates, fats, vitamins, and minerals) to maintain daily nutritional requirements. Such approaches offer limited immunological benefits and do not adequately address the underlying metabolic and inflammatory disturbances in cancer patients (6).

In recent years, growing attention has been directed toward the role of immunonutrition in reducing the incidence and severity of RIOM. By supplementing specific immunonutrients, this approach may help to break the vicious cycle linking nutrition, inflammation, metabolism, and immunity in cancer patients (7, 8). Immunonutrition refers to a nutritional support strategy that modulates key pathophysiological components of RIOM (such as the inflammatory amplification, mucosal injury, oxidative stress, and immune dysregulation) through the administration of specific nutrients or nutrient combinations, including glutamine, arginine, ω-3 polyunsaturated fatty acids (ω-3 PUFAs), and nucleotides (9, 10). This narrative review comprehensively summarizes the mechanisms of action and recent clinical advances of commonly used immunonutrients in the management of RIOM. It also aims to provide a basis for the rational application of immunonutrition in the comprehensive management of RIOM, with the ultimate goal of improving patient quality of life and clinical outcomes.

## 2 Literature search strategy

A literature search was conducted using PubMed and Web of Science for articles published between 2012 and 2026. The search terms included combinations of the following keywords: “head and neck cancer,” “radiation-induced oral mucositis,” “RIOM,” “immunonutrition,” “immunonutrients,” “nutritional support,” “glutamine,” “arginine,” “ω-3 PUFAs,” “omega-3 polyunsaturated fatty acids,” “nucleotides,” and “probiotics.” Articles were primarily selected based on their relevance to the topic, with priority given to peer-reviewed clinical trials, systematic reviews, meta-analyses, observational studies, and relevant preclinical studies published in English. Studies were excluded if they were not directly related to head and neck cancer, radiotherapy- or chemoradiotherapy-induced oral mucositis, or nutritional/immunonutritional interventions, or if they were conference abstracts, editorials, letters, or non-English publications without sufficient accessible data.

## 3 Common immunonutrients and their mechanism of action and application in RIOM

**Figure 1:**
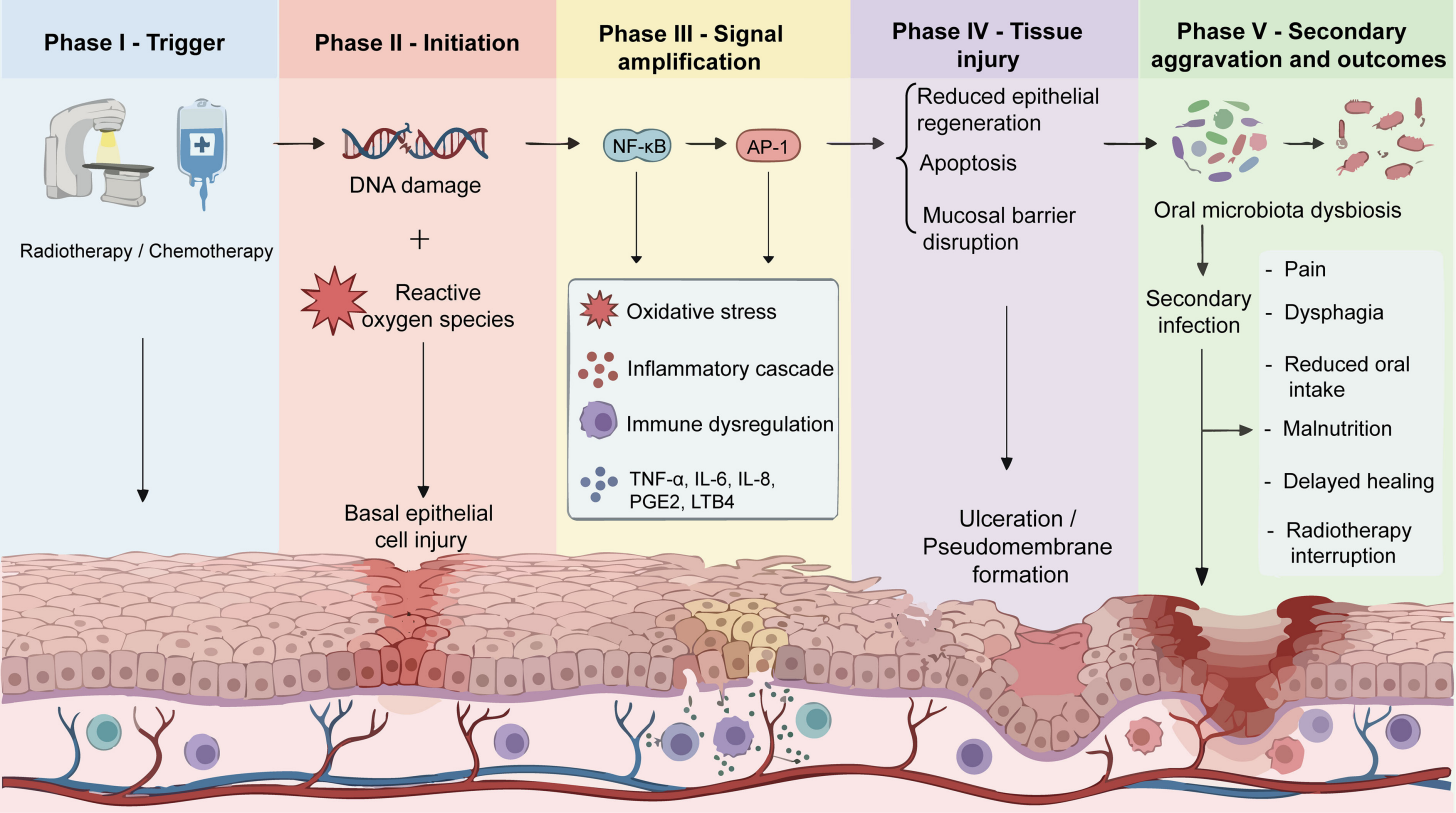
Pathobiological progression of radiation-induced oral mucositis.

**Figure 2:**
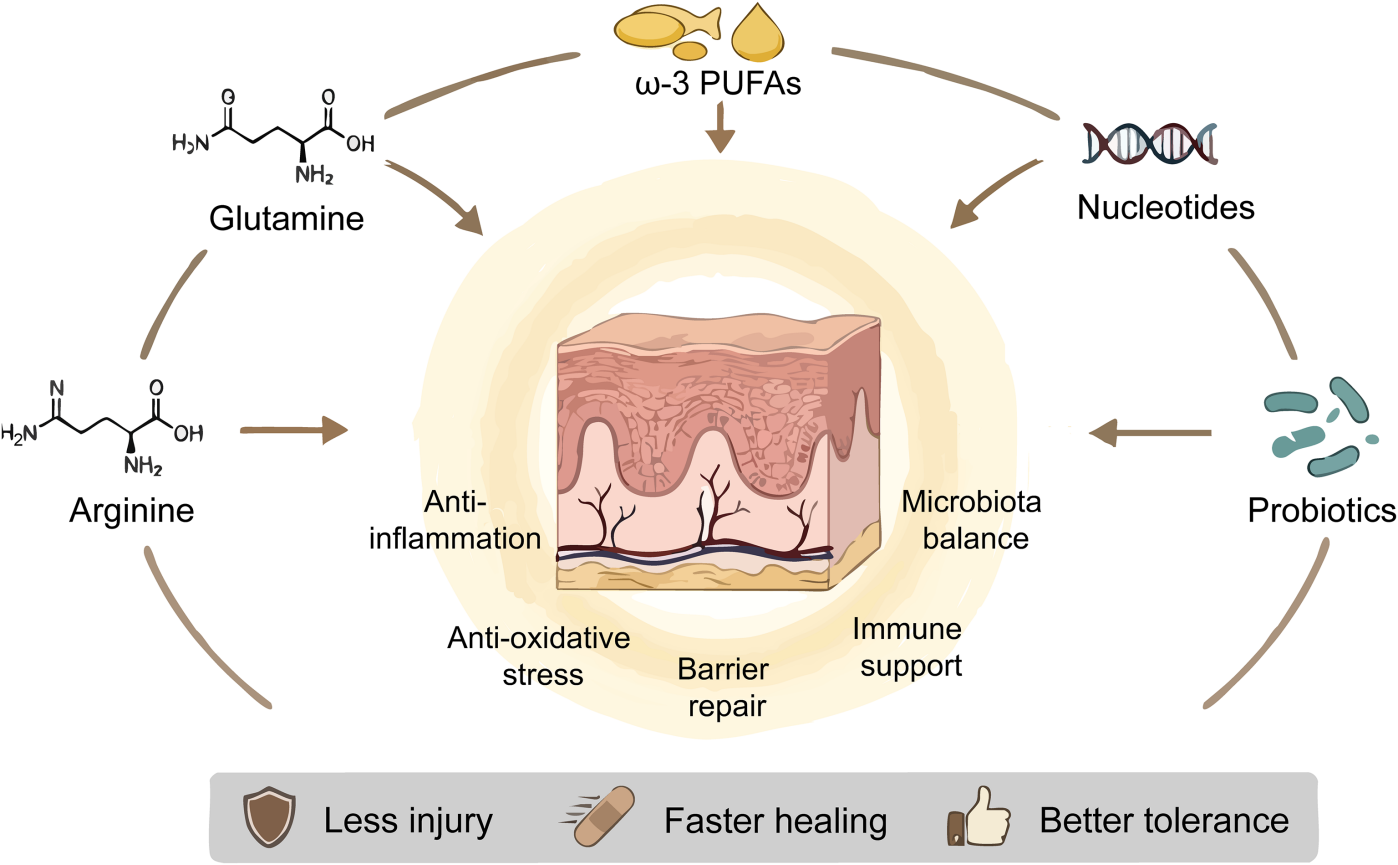
Overview of immunonutrition-mediated protection against radiation-induced oral mucositis.

The multistage pathobiological progression of RIOM is summarized in **Figure 1**. Based on this process, the potential mechanisms by which immunonutrients may contribute to the prevention and management of RIOM are summarized in **Figure 2**.

### 3.1 Glutamine

**Figure 3:**
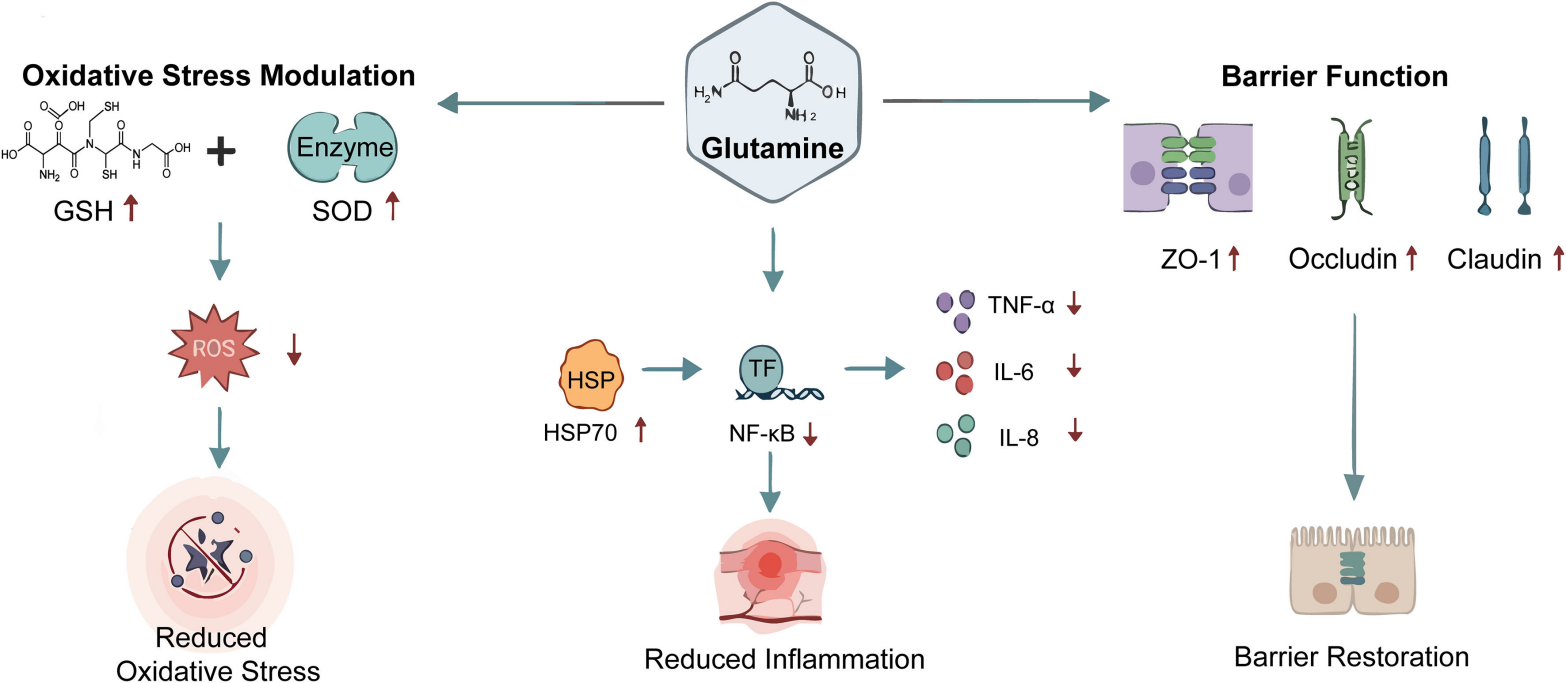
Potential mechanisms of glutamine in mitigating radiation-induced oral mucositis.

Glutamine is the most abundant free amino acid synthesized by the human body and is traditionally classified as a nonessential amino acid; however, under conditions of metabolic stress, it becomes conditionally essential (11). It plays a pivotal role in maintaining nitrogen balance and serves as a precursor for the synthesis of amino acids, proteins, nucleotides, and various biomolecules. In addition, glutamine provides an important energy source for intestinal epithelial cells, lymphocytes, neutrophils, and other cells (12). Research indicates that adequate intracellular glutamine levels enhance the detoxification and recovery capacity of normal tissues against oxidative damage caused by radiation and chemotherapy (13). Mechanistically, glutamine inhibits the nuclear factor κB (NF-κB) pathway by increasing heat shock protein 70 expression, affecting signaling pathways such as protein kinase and extracellular signal-regulated kinase. This leads to reduced production of proinflammatory cytokines, including tumor necrosis factor-α, interleukin-6 (IL-6), interleukin-8 (IL-8), thereby attenuating inflammatory responses (14). Under conditions of prolonged severe stress (e.g., burns, inflammatory bowel disease, or malignancy), endogenous glutamine synthesis is insufficient to meet physiological demands (15). Exogenous glutamine supplementation can promote the synthesis of glutathione, a key intracellular antioxidant, thereby reducing inflammation and oxidative stress. This contributes to immune protection and facilitates mucosal repair (16, 17). The potential protective mechanisms of glutamine in RIOM are summarized in **Figure 3**. Moreover, some studies have suggested that glutamine may influence glutathione metabolism in tumor cells. However, given that glutamine metabolism in cancer is highly complex and context-dependent, its effects on the antioxidant capacity and survival of cancer cells remain debated (18). Therefore, further mechanistic and clinical studies are needed. Preclinical studies support the protective role of glutamine in RIOM. Dvoretskiy S et al. (19) found that in a hamster model of induced RIOM, the treatment group administered a glutamine-containing amino acid/metabolite mixture exhibited lower mucositis scores (≥3) at least two consecutive time points during the study period compared to the control group, with a significant reduction in the number of days with severe RIOM in the treatment group. Clinical evidence further corroborates these findings. A 2021 meta-analysis synthesizing 16 randomized controlled trials (RCTs) reported that glutamine significantly reduced the risk of severe (grade 3–4) RIOM by 25%, with an even greater 49% reduction observed in preventive settings (20). Additionally, a pooled analysis of 11 RCTs involving 922 patients demonstrated that glutamine supplementation significantly decreased the severity and incidence of RIOM, reduced opioid analgesic use, and lowered the rates of nasogastric tube feeding and treatment interruption (13). Regarding the duration of administration, clinical evidence suggests that glutamine supplementation should be initiated on the first day of radiotherapy and maintained continuously throughout the entire treatment course, which typically lasts for 6 to 7 weeks (21, 22). Collectively, both preclinical and clinical evidence indicate that glutamine may delay the onset of RIOM, mitigate its severity, and promote mucosal healing.

### 3.2 Arginine

**Figure 4:**
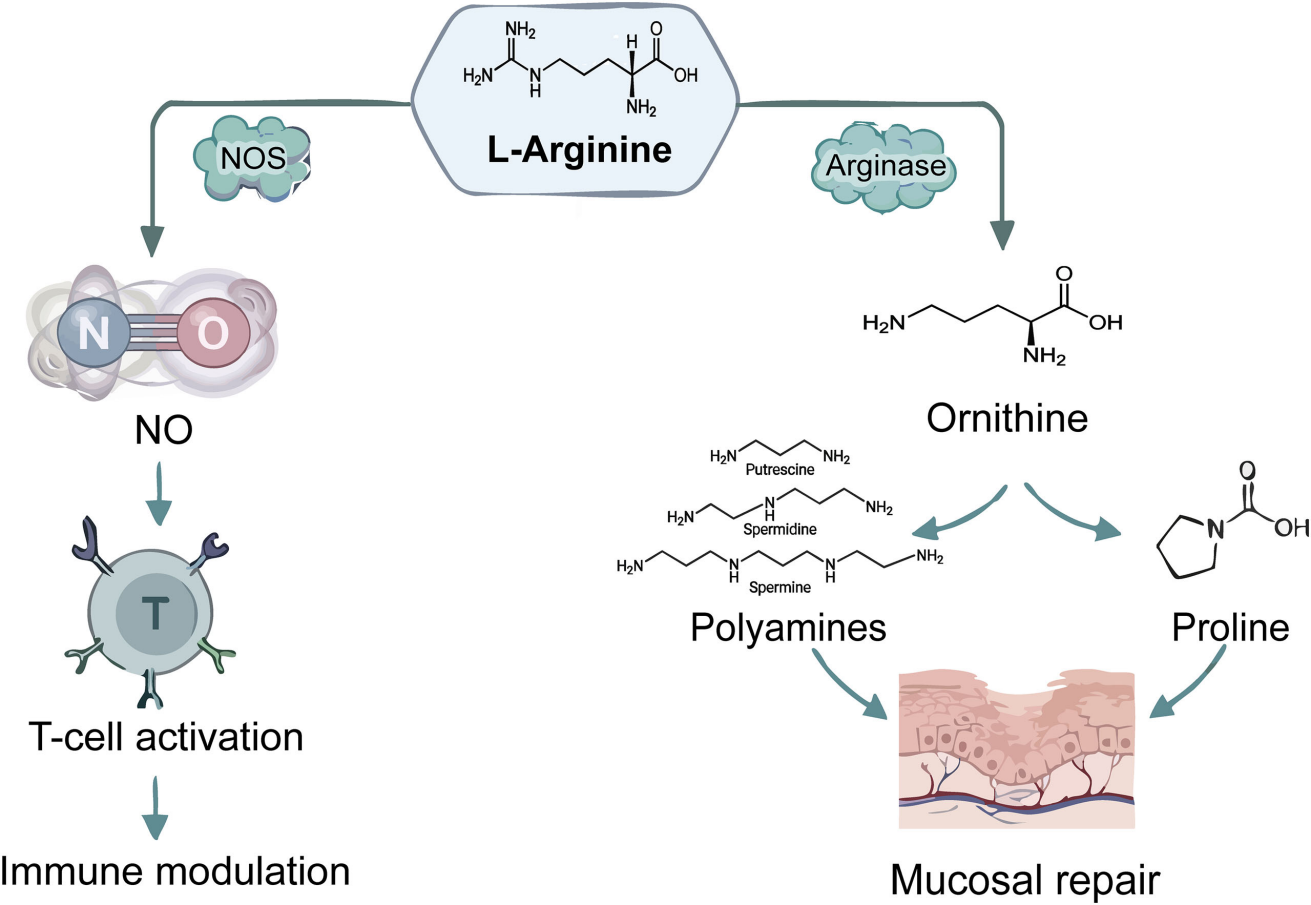
Arginine-related metabolic pathways in immune modulation and mucosal repair.

Arginine plays a crucial role in the urea cycle and serves as a precursor for a variety of essential biomolecules, including proteins, polyamines, creatine, and proline (23). It has been shown to enhance immune function and contribute to wound healing and inflammation control as summarized in **Figure 4**. Arginine is the sole source of nitric oxide (NO) synthesis in the body, promoting NO production via NO synthase; NO then activates the p53 gene and inhibits cyclin-dependent kinases, playing a critical role in mediating T-cell proliferation and immune function, ultimately leading to tumor cell apoptosis (24). In addition, arginine has been reported to inhibit tumor proliferation and metastasis, enhance postoperative recovery, and reduce the incidence of postoperative complications (25). De Luis DA et al. (26) enrolled 84 patients with oral and laryngeal cancer and observed a trend toward shorter postoperative hospital stays with supplementation of 18.9 g/day arginine. Pan Yu et al. (27) enrolled 86 malnourished nasopharyngeal carcinoma patients undergoing radiotherapy and found that those receiving arginine-supplemented individualized nutritional support exhibited significantly smaller declines in hemoglobin levels, lymphocyte counts, and body weight. Additionally, this group showed a lower incidence of oral mucositis, reduced treatment interruption rates, and higher treatment completion rates, indicating improved nutritional status and reduced treatment-related toxicity. Furthermore, in a triple-blind randomized controlled trial, the arginine group demonstrated significantly reduced RIOM severity, pain, and weight loss compared to the maltodextrin group, while improving quality of life in HNC patients (28). Regarding the administration duration, in available clinical studies, arginine supplementation was commonly initiated on the first day of radiotherapy and continued until completion of the radiotherapy course (29). Overall, these findings suggest that arginine may help reduce the incidence and severity of RIOM and improve treatment tolerance in patients with HNC. However, existing studies are constrained by relatively small sample sizes, heterogeneous intervention protocols, and insufficient long-term follow-up. Therefore, the optimal dose, timing, duration, and target population for arginine supplementation remain to be clarified in future well-designed clinical trials.

### 3.3 Omega-3 polyunsaturated fatty acids

**Figure 5:**
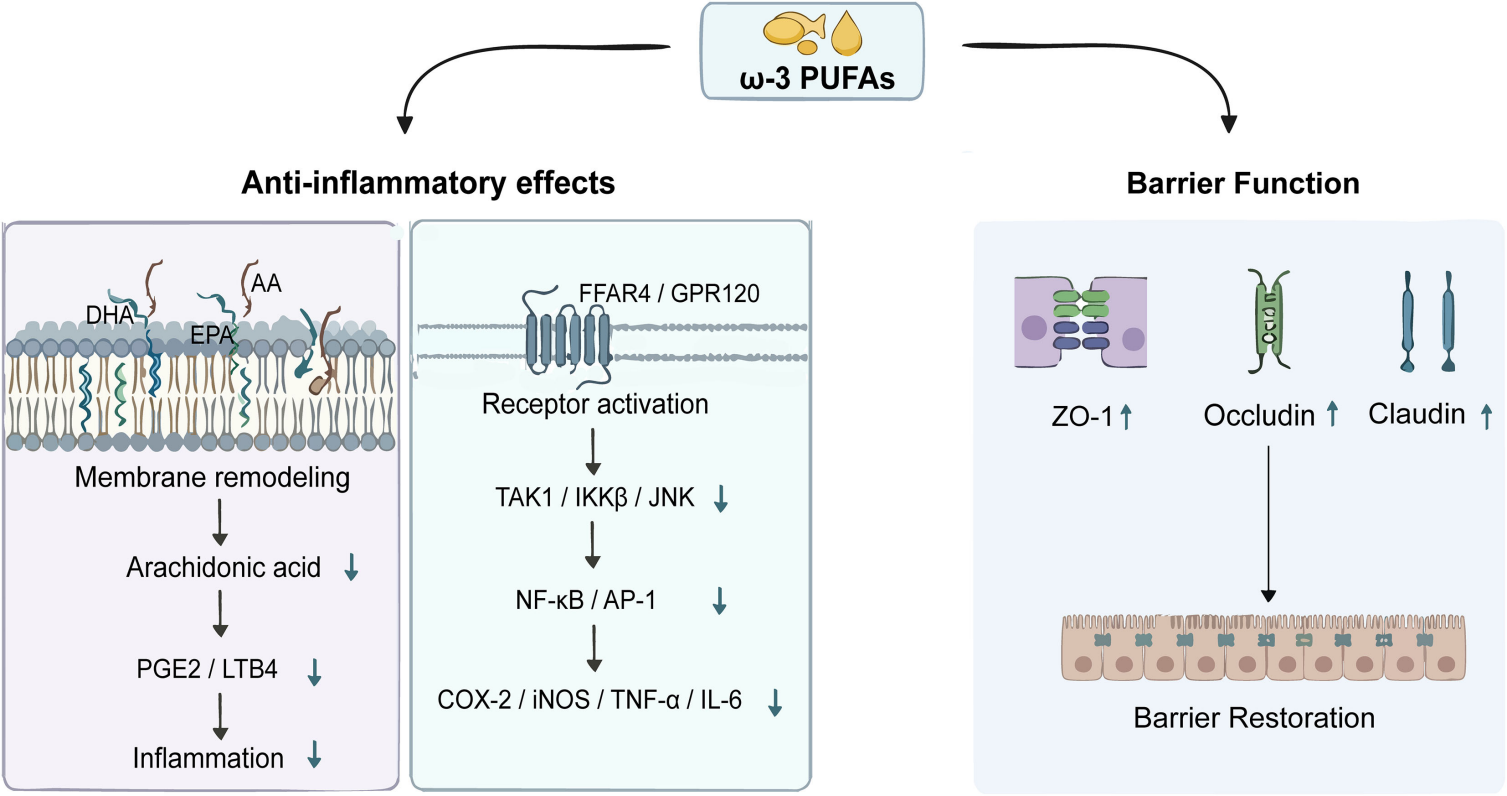
Anti-inflammatory and barrier-supportive mechanisms of ω-3 polyunsaturated fatty acids.

The ω-3 PUFAs are essential fatty acids that cannot be synthesized endogenously and must be obtained through dietary intake (30). Among them, docosahexaenoic acid (DHA) and eicosapentaenoic acid (EPA) are the most biologically active forms. These fatty acids influence cell migration, receptor binding, and receptor-ligand interactions, thereby modulating cytokine production and exhibiting anti-inflammatory effects (31). The anti-inflammatory mechanisms of ω-3 PUFAs are multifaceted. They can displace arachidonic acid from cell membranes to influence the production and release of various inflammatory mediators; enhance macrophage phagocytosis and leukocyte function; and modulate key transcription factors such as NF-κB and activator protein-1, leading to the regulation of immune responses. In addition, ω-3 PUFAs reduce the production of prostaglandin E2 and leukotriene B4 to decrease inflammatory responses (31, 32). **Figure 5** summarizes the anti-inflammatory and barrier-supportive mechanisms of ω-3 PUFAs. Beyond their anti-inflammatory properties, ω-3 PUFAs have demonstrated antitumor effects, including the promotion of tumor cell apoptosis, inhibition of tumor cell growth and metastasis, enhancement of radiotherapy sensitivity, and potential reductions in cancer incidence and mortality (33). Both clinical and animal studies have suggested that ω-3 PUFAs confer benefits in various oral inflammatory conditions, such as gingivitis, periodontal disease, and recurrent aphthous stomatitis (34–36). In a randomized, double-blind trial involving 38 cancer patients, a one-week fish oil-enriched dietary intervention significantly increased EPA and DHA incorporation into leukocytes. This was accompanied by a decrease in serum prostaglandin E2 levels in the intervention group, whereas levels increased in the control group, indicating a systemic anti-inflammatory effect of ω-3 PUFAs during radiotherapy (37). More recently, a 2023 randomized clinical trial evaluated the preventive effect of a topically applied ω-3 nanoemulsion gel in HNC patients undergoing radiotherapy. The results showed that patients receiving the ω-3 nanoemulsion gel exhibited significantly lower mucositis severity and reduced pain scores at 6 weeks post-radiotherapy compared with controls (38). These findings are consistent with the role of ω-3 PUFAs in maintaining epithelial integrity and promoting wound healing, partly through the preservation of tight junctions and reduction of cell necrosis (39). Collectively, current evidence supports both systemic and topical applications of ω-3 PUFAs as promising strategies for the prevention and management of RIOM.

### 3.4 Nucleotides

**Figure 6:**
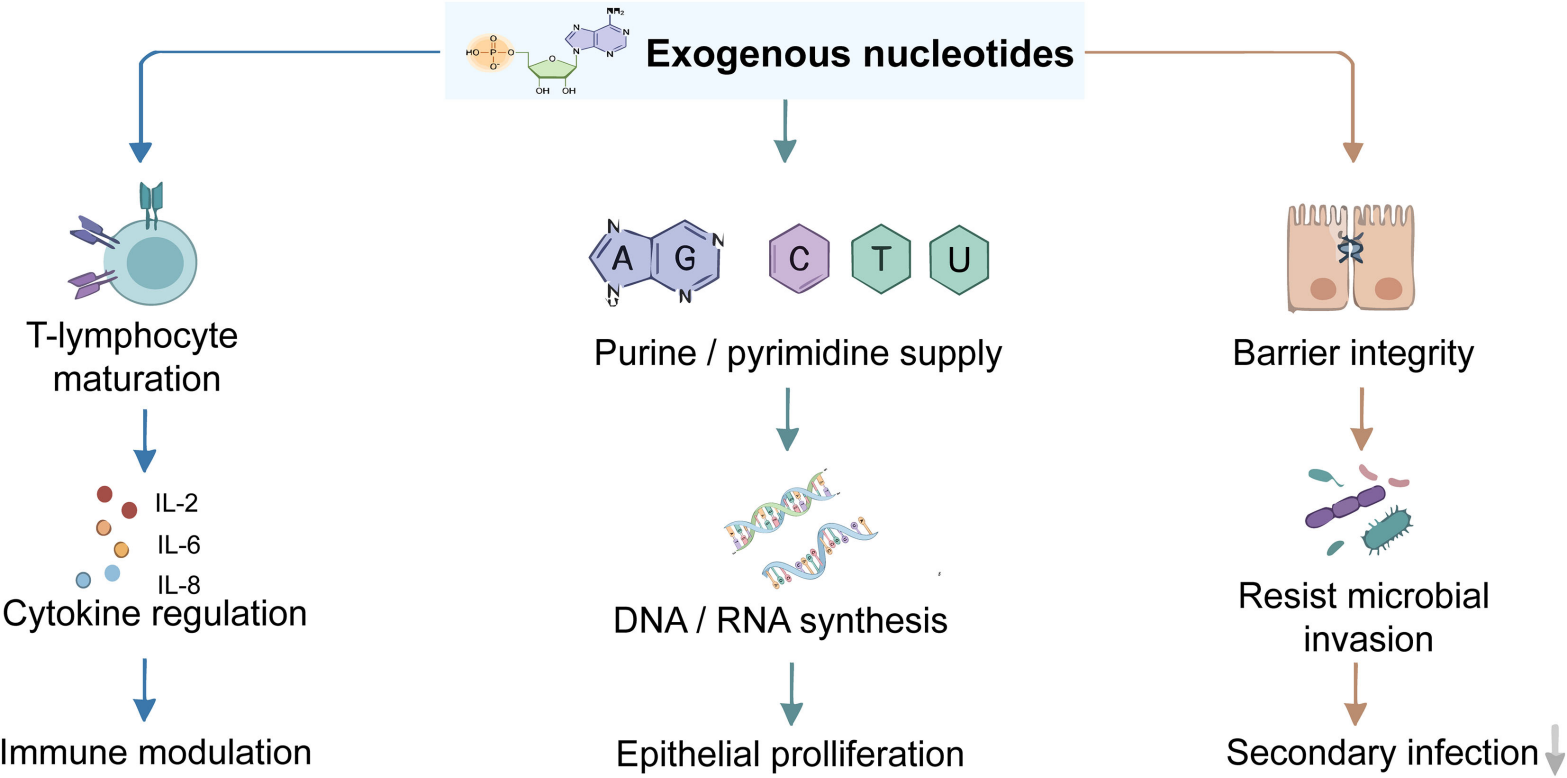
Proposed roles of exogenous nucleotides in immune regulation and epithelial repair.

Nucleotides are essential small molecules composed of purines or pyrimidines, ribose or deoxyribose, and phosphate. They participate in nearly all major biochemical reactions within the body, contributing to cellular energy transfer and nutrient metabolism, and also play important roles in immune regulation (40). As depicted in **Figure 6**, nucleotide availability has been shown to influence T-lymphocyte maturation and cytokine production, including interleukin-2, IL-6, and IL-8 (41). Although nucleotides can be synthesized endogenously, this capacity may become insufficient under conditions of physiological stress, such as severe illness or malnutrition. In such situations, dietary nucleotide supplementation may help support immune function and facilitate tissue repair (42). In surgical oncology, nucleotide-enriched diets have been associated with reduced postoperative complications and shorter hospital stays (43). Under radiation-induced stress, the demand for nucleotides increases substantially, while the capacity of the *de novo* synthesis pathway becomes limited (44). Exogenous nucleotide supplementation can promote mucosal epithelial regeneration by providing purine and pyrimidine substrates, thereby enhancing mucosal barrier integrity and immune defense (45). Vasson MP et al. (46) found that cancer patients receiving enteral nutrition during radiotherapy showed a significant increase in body weight, along with improvements in serum albumin levels, nutritional indices, and plasma antioxidant capacity, suggesting enhanced nutritional and functional status. In addition, experimental studies have demonstrated that nucleotide supplementation alleviates mucosal injury and reduces bacterial and endotoxin translocation in models of intestinal endotoxin damage (47). In the context of oral mucositis, nucleotides may promote epithelial repair and maintain mucosal integrity, thereby reducing the risk of secondary infection and contributing to improved clinical outcomes. However, direct clinical evidence for nucleotide supplementation specifically in RIOM remains limited. At present, its potential role is supported mainly by mechanistic rationale and indirect evidence related to epithelial repair, immune modulation, and intestinal barrier function.

### 3.5 Probiotics

**Figure 7:**
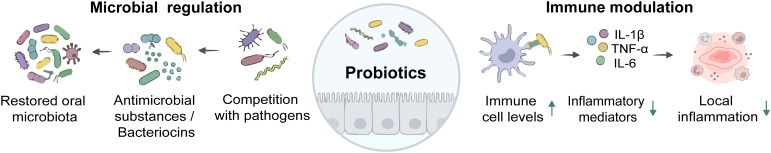
Probiotic-mediated modulation of oral microbiota and mucosal immunity.

Probiotics are live microorganisms and their bioactive components that confer health benefits when administered in adequate amounts, particularly in regulating disease-associated dysbiosis (43). Evidence suggests that probiotics help maintain microecological balance by producing antimicrobial substances, competing with pathogens for nutrients and adhesion sites, and modulating host immune responses, thereby exhibiting anti-inflammatory and immunoregulatory effects (48). Increasing evidence indicates that radiotherapy induces dynamic alterations in the oral microbiota. For example, overgrowth of Gram-negative bacteria and the associated release of endotoxins have been shown to exacerbate the severity of mucositis in patients with nasopharyngeal carcinoma undergoing radiotherapy (49). In this context, probiotics may help restore microbial homeostasis and regulate local inflammatory responses in patients with HNC (50, 51). Jiang et al. (52) conducted a randomized double-blind trial demonstrating that probiotic supplementation reduced the incidence and severity of oral mucositis, elevated immune cell levels (e.g., CD3, CD4, CD8 T cells, and lymphocytes), promoted gut microbial diversity, and enhanced the efficacy of radiotherapy. Wang Y et al. (53, 54) found that treatment with the probiotic S. salivarius K12 alleviated the onset of RIOM and restored oral microbiota balance in mice, with additional clinical evidence suggesting similar benefits in patients undergoing radiotherapy for head and neck tumors. Additionally, another randomized, double-blind study found that administration of *Lactobacillus brevis* CD2 lozenges reduced both the severity and incidence of chemoradiotherapy-induced mucositis in HNC patients. The incidence of mucositis was 52% in the treatment group compared with 77% in the control group, and treatment completion rates were correspondingly improved (55). Notably, probiotics in these studies were administered orally and may exert indirect effects on RIOM by modulating systemic immune responses and stabilizing gastrointestinal microbiota (**Figure 7**). However, the precise mechanisms underlying their protective effects in oral mucositis remain to be fully elucidated, and further well-designed studies are warranted.

### 3.6 Other proposed nutrients and natural agents

Although classic immunonutrients form the basis of dietary interventions, attention has also been directed toward certain promising trace elements and natural substances as potential adjunctive strategies for RIOM management. Zinc is an essential trace element involved in tissue repair and antioxidant defense; it promotes wound healing and supports immune function (56). Three RCTs included in a previous meta-analysis showed that oral zinc sulfate supplementation may reduce the incidence and severity of RIOM in patients with HNC (57). Similarly, curcumin, a natural polyphenolic compound with potent anti-inflammatory properties, has attracted attention for its potential role in the management of oral inflammation (58). A randomized, double-blind, controlled trial evaluated the effects of a bio-enhanced turmeric formulation on RIOM. The results showed that only 25% and 20% of patients in the BTF 1 g/day and 1.5 g/day arms developed grade 3 oral mucositis, respectively, compared with 65% of patients in the placebo arm (59). In addition, honey is a natural product with wound-healing properties that may stimulate saliva secretion and modulate immune responses (60). It exerts anti-inflammatory and antioxidant effects by reducing prostaglandin levels and increasing nitric oxide levels at the site of injury, thereby accelerating the healing of acute and chronic wounds (61). A systematic review and meta-analysis conducted by Tian et al. (62) found that honey provides clinical benefits for RIOM. A recent meta-analysis, which included 10 studies involving a total of 599 patients undergoing radiation therapy, showed that the prophylactic use of honey-either topically or orally-significantly reduced the severity of RIOM in HNC patients, decreased the need for potent analgesics, and prevented excessive weight loss (63). Overall, although these agents have not yet been shown to possess the systemic immunomodulatory effects of composite immunonutritional supplements, they are highly accessible, cost-effective, and well-tolerated as adjunctive strategies for improving patients’ quality of life.

## 4 Clinical application and advantages of composite immunonutrients

Given the multifactorial etiology and complex pathophysiological mechanisms of RIOM, single-nutrient interventions are often insufficient to comprehensively target the interconnected pathways involved, including inflammation, oxidative stress, immune dysregulation, and mucosal barrier injury (64). In contrast, composite immunonutrient formulations, which combine multiple nutrients with synergistic or complementary functions, can simultaneously modulate multiple signaling pathways. Moreover, composite formulations can better address the nutritional deficiencies commonly observed in cancer patients undergoing radiotherapy, helping to maintain energy balance, preserve lean body mass, and improve treatment tolerance and adherence (65). As a result, multi-nutrient strategies are playing an increasingly important role in the nutritional management of RIOM.

### 4.1 Commonly used compound formulation components

Common oral or enteral immunonutrient formulations typically include the following components (41): (1) Core immunonutrients: glutamine, arginine, ω-3 PUFAs, nucleotides; (2) Macronutrient support: adequate energy supply (carbohydrates, fats, proteins/amino acids). Protein sources are often provided as hydrolyzed proteins or free amino acids to enhance absorption; (3) Micronutrients: vitamins (particularly antioxidant vitamins A, C, E, D, and B-complex), minerals (e.g., zinc, selenium—involved in antioxidant enzyme synthesis), trace elements; (4) Additional functional components: dietary fiber (prebiotic effect), antioxidants (e.g., N-acetylcysteine, flavonoids), specific amino acids (e.g., branched-chain amino acids).

### 4.2 Latest clinical application research

The European Society for Clinical Nutrition and Metabolism (ESPEN) practice guidelines recommend that malnourished patients undergoing major oncological surgery receive specialized nutritional formulations enriched with arginine, ω-3 PUFAs, and ribonucleotides during the perioperative period or at least postoperatively (66). During radiotherapy or chemoradiotherapy, 55% of patients may experience additional weight loss of 10% or more, and the deterioration of nutritional status can lead to treatment interruption or discontinuation. In this context, immunonutrition has been shown to reduce the risk of treatment interruption and improve treatment tolerance (67). Machon C et al. (68) enrolled 31 patients with non-metastatic stage III or IV head and neck squamous cell carcinoma undergoing concurrent radiotherapy. Patients received an oral nutritional supplement containing amino acids, ω-3 PUFAs, ribonucleic acid, vitamins, and antioxidants for five days prior to each chemotherapy cycle. The results showed improved inflammatory status and a reduced incidence of severe acute mucositis. Immunonutrition can improve nutritional status and reduce weight loss. Similarly, Valentini V et al. (69) provided immunonutrition along with dietary counseling to 21 patients with HNC undergoing radiotherapy over a three-month period. Compared with historical data, patients exhibited reduced body weight loss and a lower incidence of grade 3–4 oral mucositis, suggesting that oral nutritional supplements (ONS) may help maintain nutritional status and mitigate RIOM severity. A randomized controlled trial published in 2024 compared early versus delayed nutritional intervention in patients undergoing radiotherapy for HNC. The results showed that the incidence of grade 3–4 oral mucositis was only 2% in the early intervention group, compared to 14% in the late intervention group. Additionally, patients receiving early intervention experienced less weight loss by week 7 of radiotherapy, and demonstrated significantly better nutritional status as assessed by the Patient-Generated Subjective Global Assessment (PG-SGA) (70). Although several studies have suggested potential benefits of immunonutrition in improving nutritional status, inflammatory profiles, or treatment tolerance in patients with head and neck cancer undergoing radiotherapy or chemoradiotherapy, the evidence regarding its effect on RIOM prevention remains inconsistent. Notably, the phase III randomized controlled trial by Boisselier et al. (10) reported that an immunomodulating nutritional formula did not significantly reduce the incidence of severe oral mucositis compared with the control formula. Similarly, Dechaphunkul et al. (8) found no significant difference in the incidence of grade 3–4 oral mucositis between the immunonutrition and control groups. These findings suggest that immunonutrition should not be presented as uniformly effective for RIOM prevention. One possible explanation is that, in some trials, the duration or timing of supplementation may have been insufficient to counteract the cumulative mucosal injury caused by daily radiotherapy. Therefore, while immunonutrition may provide nutritional and supportive benefits in selected patients, further well-designed prospective studies are needed to clarify its specific role in preventing or reducing RIOM. The key clinical studies evaluating immunonutritional interventions for RIOM in patients with HNC receiving radiotherapy or chemoradiotherapy are summarized in **Table 1**.

**Table 1:** Clinical trials of nutritional and immunonutritional interventions for radiation-induced oral mucositis. .

Journal/year	Study design	Population/sample size	Treatment mode	Start time before RT	Duration	Control group	Oral mucositis incidence/severe RIOM	Core abstract/figure-ready takeaway	Ref.
Head & Neck, 2023	Retrospective cohort study	Nasopharyngeal carcinoma (n=161)	CCRT	At the start of CCRT	Throughout CCRT	routine ONS	Early ONS could reduce the rate of weight loss and grade 3 RIOM, as well as improve treatment adherence.	Early nutritional intervention may improve nutritional outcomes and protect against severe RIOM.	(5)
Clinical Nutrition, 2022	Phase II randomized, double-blind study	Head and neck cancer (n=110)	CCRT	5 consecutive days before each chemotherapy session	Before each chemotherapy cycle during CCRT	Isocaloric and isonitrogenous control formula	Grade 3–4 oral mucositis: 62% in immunonutrition group vs 67% in control group (P = 0.690)	Immunonutrition did not significantly reduce severe oral mucositis during definitive CCRT, but showed a trend toward better 3-year PFS and OS, suggesting potential benefits beyond RIOM prevention.	(8)
American Journal of Clinical Nutrition, 2020	Double-blind phase III randomized trial	Head and neck cancer (n=172)	Postoperative adjuvant CCRT	5 days before each chemotherapy cycle	Short repeated courses before each chemotherapy cycle during CCRT	Isoenergetic/isonitrogenous control formula	Severe OM: 33.7% vs 34.9% (P = 0.872)	The combined immunonutrition formula did not significantly reduce severe OM, but it is high-quality randomized evidence showing that findings are not fully consistent.	(10)
Clinical Nutrition ESPEN, 2023	Prospective randomized open-label phase II study	Head and neck squamous cell carcinoma (n=75)	Platinum-based CCRT	The first day of CRT	During PBCRT	The control group received an isocaloric isonitrogenous standard enteral nutrition formula.	Grade 3 mucositis was 25% in HMB/Arg/Gln group vs 64.6% in non-intervention group (P = 0.0003).	HMB/Arg/Gln significantly inhibited progression to grade 3 mucositis in the late phase of PBCRT and reduced body weight loss.	(17)
Clinical Nutrition, 2014	Randomized double-blind clinical trial	Head and neck cancer and esophageal cancer (n=37)	Radio-chemotherapy	5 days before RCT	5 days before and until the end of RCT (5–7 weeks)	Nitrogenous, isoenergetic Standard Enteral Nutrition (SEN)	No significant difference was observed for mucositis despite a higher proportion of patients presenting severe mucositis (grade 3) at the end of RCT in SEN patients (7/13) than in IEN patients (6/15).	The main signal is improved nutritional and functional status; evidence for RIOM improvement is limited.	(46)
Supportive Care in Cancer, 2012	Prospective clinical study	Non-metastatic stage III/IV head and neck squamous cell carcinoma (n=31)	Concurrent radio-chemotherapy	5 days before each chemotherapy cycle	Throughout RCT	Before–after comparison	Severe acute mucositis: 16% (5/31, NCI grade 3–4)	Short-course prophylactic combined immunonutrition may improve inflammatory/oxidative-stress status and may reduce severe acute mucositis.	(68)

RT, radiotherapy; CRT, chemoradiotherapy; CCRT, concurrent chemoradiotherapy; RCT, radio-chemotherapy; PBCRT: Platinum-Based Concurrent Chemoradiotherapy; HMB, β-hydroxy-β-methylbutyrate; Arg, arginine; Gln, glutamine; ONS, Oral Nutritional Supplements; RIOM, Radiation-induced oral mucositis; PFS, Progression-Free Survival; OS, Overall Survival OM, Oral Mucositis; SEN, Standard Enteral Nutrition; NCI, National Cancer Institute.

### 4.3 Timing and strategies for application

(1) Prophylactic Application: Providing immunonutritional support before or during the initiation and amplification phases of mucosal injury is more conducive to enhancing mucosal barrier resistance, suppressing initial inflammatory responses, and maintaining immune function (71). (2) Therapeutic Application: Once severe ulceration has developed and a vicious cycle of inflammation and infection is established, nutritional intervention may still promote tissue repair and alleviate symptoms, but complete reversal of mucosal damage becomes more difficult. This approach is suitable for patients without prevention or with failed prevention (72); (3) Continuous Application: To improve cancer patients’ tolerance to radiotherapy and chemotherapy and reduce treatment-related adverse reactions, continuous application throughout the peri-radiotherapy period is recommended, with appropriate extension after treatment completion based on mucositis recovery status (73).

### 4.4 Individualized nutritional assessment and plan development

Currently, the most commonly used nutritional risk screening tools in clinical practice are the Nutritional Risk Screening 2002 (NRS 2002), the Malnutrition Universal Screening Tool, the Mini Nutritional Assessment, and the PG-SGA. Among these, the NRS 2002 and PG-SGA scales are recommended by multiple domestic expert consensus statements as screening and assessment tools for nutritional risk in patients undergoing radiotherapy for malignant tumors (74, 75). Beyond these nutritional scoring tools, comprehensive nutritional status analysis may also incorporate indicators such as body mass index, triceps skinfold thickness, upper arm muscle circumference, serum albumin levels, and lymphocyte counts (75). Notably, early initiation of nutritional support, preferably from the time of cancer diagnosis and before the start of radiotherapy, may be more beneficial than delayed or reactive intervention. In clinical practice, patients should be encouraged to obtain adequate energy, protein, and immunonutrients through a balanced diet whenever possible, including high-quality protein sources, omega-3-rich foods, and other nutrient-dense foods (66). When oral intake is insufficient, ONS or enteral nutrition should be considered according to the patient’s nutritional status, swallowing function, gastrointestinal tolerance, lifestyle, dietary habits, comorbidities, and treatment-related symptoms (76). In the clinical implementation of immunonutrition support therapy, the five-step treatment principle for malnutrition is primarily followed: dietary and nutritional education, ONS, total enteral nutrition, a combination of enteral and parenteral nutrition, and total parenteral nutrition. Treatment should proceed step by step, prioritize enteral nutrition, and be individualized. When 60% of the target energy requirement cannot be met for 3–5 days, dynamic assessment and timely adjustment should be carried out to achieve the patient’s nutritional goals (77).

Formulation selection includes: (1) Oral preparations (liquids, powdered drinks): Suitable for patients capable of ingesting sufficient amounts orally (78). (2) Enteral nutrition preparations (tube feeding): Suitable for patients with severe dysphagia, significant oral intake insufficiency, or requiring nasogastric feeding/percutaneous endoscopic gastrostomy (76). (3) Parenteral nutrition support: Suitable for patients with severe intestinal dysfunction who cannot tolerate enteral nutrition. Patients receiving radiotherapy or chemoradiotherapy who consume <60% of target daily energy intake for over 10 consecutive days should receive supplemental parenteral nutrition (79). (4) Nanoemulsion technology: Enhances bioavailability of liposoluble components (e.g., ω-3 PUFAs) and improves local mucosal permeability (38).

## 5 Current challenges and directions

Despite significant advances in immunonutrition for the prevention and treatment of RIOM, numerous challenges remain. First, there is still a lack of long-term, multicenter, large-sample RCTs targeting novel single components (such as specific probiotic strains or novel antioxidants), innovative formulations (like topical sustained-release patches), and optimized combination regimens. More comparative studies directly evaluating different immunonutrition strategies are needed (80). Furthermore, while multiple assessment methods exist for RIOM, their application requires stricter standardization. Beyond objective mucositis grading, greater incorporation of patient-reported outcomes is essential. These should include pain intensity, swallowing function and nutrition-related quality of life (e.g., the quality-of-life section of PG-SGA) to comprehensively reflect intervention efficacy (1). While current literature suggests relationships between existing immunonutrient dosages and efficacy, guidelines supporting optimal dosing for established immunonutrient formulations remain lacking. Further exploration is needed, as clinical research and evaluation of immunonutrition functions currently lack unified standards.

## 6 Conclusion

RIOM poses a major challenge for patients undergoing radiotherapy for head and neck tumors, significantly impacting treatment progression and quality of life. Immunonutrient offers comprehensive protection against RIOM through “multi-targeted, networked” molecular mechanisms: they reduce harmful inflammation and oxidative stress while enhancing beneficial barrier and immune functions, thereby promoting normal tissue repair without compromising antitumor efficacy. Current evidence suggests that composite immunonutrient formulations and early, continuous intervention appear to provide greater clinical benefits. However, high-quality evidence regarding optimal formulations, dosing strategies, and timing of administration is still limited. Future well-designed, large-scale clinical studies are needed to establish standardized protocols and further define the role of immunonutrition in the management of RIOM.
